# SOX1 down-regulates β-catenin and reverses malignant phenotype in nasopharyngeal carcinoma

**DOI:** 10.1186/1476-4598-13-257

**Published:** 2014-11-26

**Authors:** Zhong Guan, Jialiang Zhang, Jing Wang, Hefei Wang, Feimeng Zheng, Jieren Peng, Yaodong Xu, Min Yan, Bing Liu, Bai Cui, Ying Huang, Quentin Liu

**Affiliations:** Department of Otorhinolaryngology, Sun Yat-sen Memorial Hospital, Sun Yat-sen University, Guangzhou, 510120 China; State Key Laboratory of Oncology in South China, Collaborative Innovation Center of Cancer Medicine, Sun Yat-sen University Cancer Center, Guangzhou, 510060 China; Institute of Cancer Stem Cell, Cancer Center, Dalian Medical University, Dalian, 116044 China

**Keywords:** SOX1, β-catenin, Methylation, Differentiation, NPC

## Abstract

**Background:**

Aberrant activation of the Wnt/β-catenin signaling pathway is an important factor in the development of nasopharyngeal carcinoma (NPC). Previous studies have demonstrated that the developmental gene sex-determining region Y (SRY)-box 1 (*SOX1*) inhibits cervical and liver tumorigenesis by interfering with the Wnt/β-catenin signaling pathway. However, the role of SOX1 in NPC remains unclear. This study investigates the function of SOX1 in NPC pathogenesis.

**Results:**

Down-regulation of SOX1 was detected in NPC cell lines and tissues. Besides, quantitative methylation-specific polymerase chain reaction revealed that SOX1 promoter was hypermethylated in NPC cell lines. Ectopic expression of SOX1 in NPC cells suppressed colony formation, proliferation and migration *in vitro* and impaired tumor growth in nude mice. Restoration of SOX1 expression significantly reduced epithelial-mesenchymal transition, enhanced cell differentiation and induced cellular senescence. Conversely, transient knockdown of SOX1 by siRNA in these cells partially restored cell proliferation and colony formation. Notably, SOX1 was found to physically interact with β-catenin and reduce its expression independent of proteasomal activity, leading to inhibition of Wnt/β-catenin signaling and decreased expression of downstream target genes.

**Conclusions:**

SOX1 decreases the expression of β-catenin in a proteasome-independent manner and reverses the malignant phenotype in NPC cells.

**Electronic supplementary material:**

The online version of this article (doi:10.1186/1476-4598-13-257) contains supplementary material, which is available to authorized users.

## Background

Nasopharyngeal carcinoma (NPC) is the most common head and neck cancer in southern China, Southeast Asia, the Arctic and the middle/northern regions of Africa. The incidence of NPC in southern China is approximately 25–50 per 100,000 persons each year, which is 100-fold higher than that in Western countries [1–3]. The poor clinical outcome of NPC is largely attributable to resistance to therapies and metastasis [4]. Therefore, new strategies for safer and more effective treatment are urgently needed [5]. The molecular mechanisms underlying the pathogenesis of NPC are incompletely defined, and an enhanced understanding of these will facilitate the development of novel therapeutics.

The family of sex-determining region Y (SRY)-box (SOX) proteins is a group of transcription factors that contain a highly conserved high-mobility group (HMG) DNA-binding domain. SOX family members play crucial roles in both embryonic and postnatal development and in stem cell regulation [6–8]. Moreover, several members of the SOX family have been implicated in cancer development [9–14]. For example, SOX10 facilitates the formation of a stable SOX10/T-cell factor (TCF)-4/β-catenin complex, subsequently contributing to tumorigenesis in hepatocellular carcinoma (HCC). Similarly, SOX9 enhances tumor growth, angiogenesis and invasion in prostate cancer. However, SOX17 inhibits canonical Wnt/β-catenin signaling and suppresses tumor growth in papillary thyroid carcinoma. Consistently, SOX1 is a tumor suppressor that is suppressed by hypermethylation of its promoter region in cervical and ovarian cancers. These findings are in accordance with the notion that hypermethylation of promoter regions of tumor suppressor genes is a major contributor to carcinogenesis [15, 16]. For example, hypermethylation of the p16^*INK4a*^ promoter leads to decreased expression of its protein in NPC, further promoting tumorigenesis [17, 18]. Additionally, aberrant promoter methylation of *CDH13*, *DLEC1*, *CHFR* and *CDH1* has been implicated in tumorigenesis [19, 20]. However, whether the methylation status of the *SOX1* promoter is involved in the development of NPC remains to be elucidated.

The canonical Wnt signaling pathway is involved in various biological processes, including embryonic development, cell proliferation and stem cell maintenance [21]. Moreover, the dysregulation of Wnt signaling is implicated in human tumorigenesis. The central element of the canonical Wnt pathway is β-catenin, which forms complexes with TCF/lymphoid enhancer factor (LEF) HMG box transcription factors to stimulate the transcription of Wnt-responsive genes including *CCND1*, *MYC* and *Cdx-1*[22]. Previous studies have shown that SOX family members regulate Wnt/β-catenin signaling through interaction with β-catenin. For example, SOX7 can suppress expression of Cyclin D1 and c-Myc via direct interaction with β-catenin, thereby inhibiting Wnt/β-catenin signaling in endometrial, prostate and colon cancers [23, 24]. Additionally, SOX1 competes with TCF/LEF by physically binding to β-catenin and therefore interfering with the activation of Wnt/β-catenin signaling in HCC [25]. Therefore, we investigated whether SOX proteins regulate Wnt/β-catenin signaling in NPC.

In this study, we demonstrate that depletion of SOX1 is responsible for the malignant phenotype of NPC. We show that recovery of SOX1 expression leads to a down-regulation of β-catenin that is independent of proteasomal activity. These new data show that SOX1 decreases β-catenin activity and reverses the malignant phenotype of NPC.

## Results

### Down-regulation of SOX1 in NPC cells and tissues is associated with promoter hypermethylation

**Figure 1:**
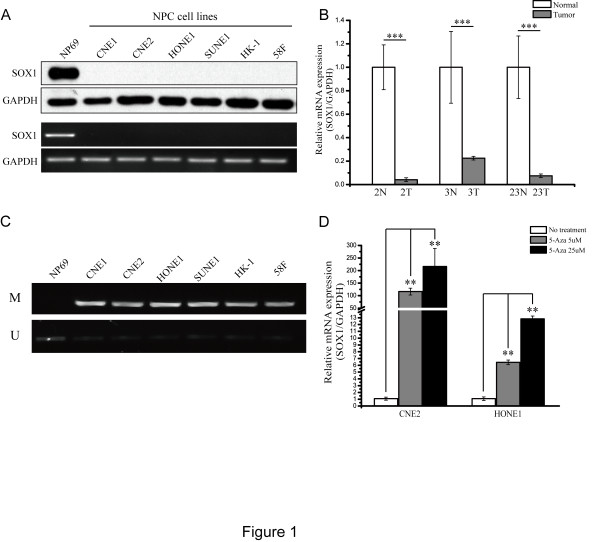
**Down-regulation of SOX1 in NPC cell lines and tissues is associated with promoter hypermethylation. (A)** Endogenous protein level (upper panel) and mRNA level (lower panel) of SOX1 were detected in NPC cell lines via WB and RT-PCR, respectively. **(B)** SOX1 transcripts of NPC tissues (T) and their corresponding adjacent non-tumor tissues (N) were determined via qRT-PCR and normalized using GAPDH expression. Data were analyzed via the ΔΔCt method and representative results from three samples (numbers 2, 3 and 23) are shown. Bar represents mean ± SD of three independent experiments (***p <0.001, Student’s t test). **(C)** Methylation status of NPC cell lines was determined by qMS-PCR. M, methylated SOX1; U, unmethylated SOX1. **(D)** NPC cell lines CNE2 and HONE1 were treated with or without 5 or 25 μM 5-Aza-CdR for 48 h. SOX1 transcripts were analyzed via qRT-PCR and normalized using GAPDH. Data were analyzed using the ΔΔCt method. Bar represents mean ± SD of three independent experiments (**p <0.01, ANOVA followed by the least significant difference test was used to make statistical comparisons).

We first examined the expression of SOX1 in six NPC cell lines and found that both mRNA and protein were barely detectable in all six cell lines. Conversely, both SOX1 mRNA and protein were highly expressed in NP69 cells, an immortalized human normal nasopharyngeal epithelial cell line (Figure 1A). We then confirmed these results in three primary NPC tissues and their corresponding adjacent non-tumor tissues using quantitative real-time polymerase chain reaction (qRT-PCR). SOX1 mRNA expression was significantly down-regulated in primary NPC tissues when compared with the adjacent non-tumor tissues (Figure 1B). We next asked whether the down-regulation of SOX1 in NPC was caused by *SOX1* promoter methylation. We determined the methylation status of the NPC cell lines by quantitative methylation-specific PCR (qMS-PCR). Hypermethylation was confirmed in the NPC cell lines that showed down-regulated SOX1 expression, whereas methylation was almost absent in NP69 cells (Figure 1C). To determine whether promoter methylation was involved in regulating SOX1, two NPC cell lines (CNE2 and HONE1) were treated with 5-AZA-2′-deoxycytidine (5-Aza-CdR), a DNA methyltransferase inhibitor. Re-expression of SOX1 was detected in both NPC cell lines when methylation was prevented (Figure 1D). These data suggest that the low levels of *SOX1* expression were attributable to promoter methylation.

### Ectopic expression of SOX1 represses NPC cells proliferation and migration

**Figure 2:**
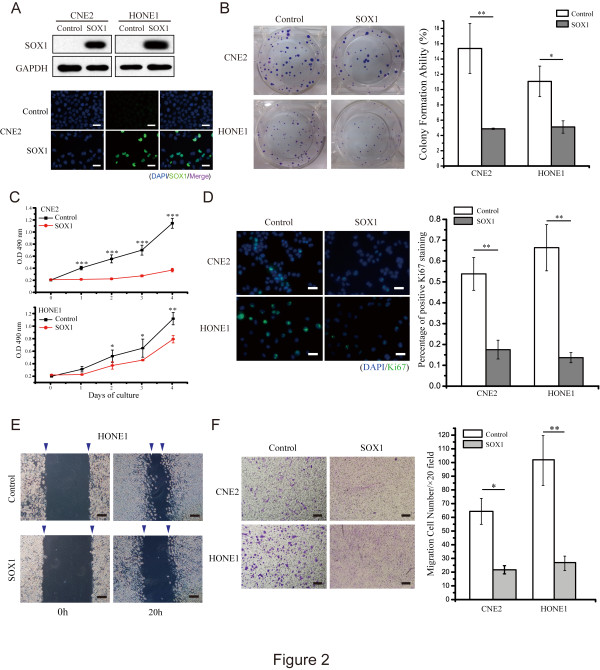
**Ectopic expression of SOX1 represses NPC cells proliferation and migration** ***in vitro*** **. (A)** Ectopic expression of SOX1 in NPC cell lines was confirmed by WB (GAPDH as internal control) and IF. **(B, C, D)** Colony formation assay, cell proliferation assay and Ki67 staining were performed in NPC cells overexpressing SOX1. The colony formation ability reduced from 15.48  ±  3.29% to 4.90  ±  0.09% in CNE2 cells and from 11.14  ±  2.01% to 5.14  ±  0.82% in HONE1 cells. The Ki67 staining rate decreased from 0.54  ±  0.08 to 0.18  ±  0.05 in CNE2 cells and from 0.66  ±  0.11 to 0.29  ±  0.02 in HONE1 cells. Bar represents mean  ±  SD of three independent experiments (*p <0.05, **p <0.01, ***p <0.001, Student’s t test) **(E, F)** Wound-healing assay and transwell migration assay were performed in NPC cells overexpressing SOX1. The transwell migration cell number for each 20× field decreased from 64.33  ±  9.5 to 21.75  ±  2.99 in CNE2 cells and from 103.0  ±  18.2 to 27.0  ±  5.30 in HONE1 cells. Quantitative data are shown as the mean  ±  SD from three independent experiments. (*p <0.05, **p <0.01, ***p <0.001, Student’s t test). (Scale bars, 50 μm in **A**, **D**, 100 μm in **E**, **F**).

Since we observed a down-regulation of SOX1 in both NPC cell lines and tissues, we next determined whether overexpression of SOX1 could reverse the malignant phenotype of NPC cells. Virus-mediated overexpression of SOX1 in CNE2 and HONE1 cells was confirmed by western blot (WB) and immunofluorescence (IF) analysis (Figure 2A). Overexpression of SOX1 significantly decreased colony formation and proliferation in both CNE2 and HONE1 cells (Figure 2B and C). SOX1 overexpression also significantly decreased the percentage of Ki67 (+) cells in both CNE2 and HONE1 cells (Figure 2D). Furthermore, we found that the migration ability of both CNE2 and HONE1 cells was significantly suppressed when SOX1 was overexpressed (Figure 2E, and F and Additional file 1: Figure S1A).

### SOX1 impairs tumor formation in a xenograft model

**Figure 3:**
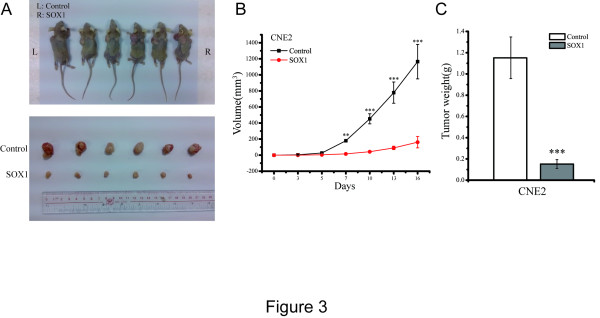
**SOX1 suppresses tumor formation in nude mice model. (A)** CNE2 cells (1 × 10^6^) virally transformed with vector-alone or SOX1 plasmid were injected into the left (vector-only) and right (SOX1) flank of nude mice, respectively (upper panel). Tumors were taken from mice of both the control group and SOX1 overexpression group after 20 days (lower panel). **(B)** Tumor growth curve of SOX1-overexpressing cells compared with that of vector-only cells. Points represent the mean tumor volumes of six independent experiments; bars represent the SD. (**p <0.01, ***p <0.001, Student’s t test) **(C)** Tumor weight from the vector-only and SOX1 groups decreased from 1.15 ± 0.20 g to 0.15 ±  0.04 g. The results were obtained from six independent experiments; bars represent the SD. (***p <0.001, Student’s t test).

Next, we examined the influence of SOX1 on NPC cells tumor formation *in vivo*. CNE2 cells stably transfected with either empty vector or SOX1 were delivered subcutaneously into nude mice and tumor growth was monitored (Figure 3A). The tumors were harvested and weighed after 20 days of growth. When compared with the control group, the mean tumor volume (Figure 3B) and tumor weight (Figure 3C) were significantly lower in mice inoculated with SOX1-overexpressing CNE2 cells. Tumor volume decreased from 1165.30 ± 205.11 mm^3^ to 161.06 ± 58.03 mm^3^ and weight decreased from 1.15 ± 0.20 g to 0.15 ± 0.04 g (both *p* <0.001). These results suggest that SOX1 impairs tumor growth in NPC cells *in vivo*.

### SOX1 overexpression reduces epithelial-mesenchymal transition, induces cell differentiation and enhances cellular senescence

**Figure 4:**
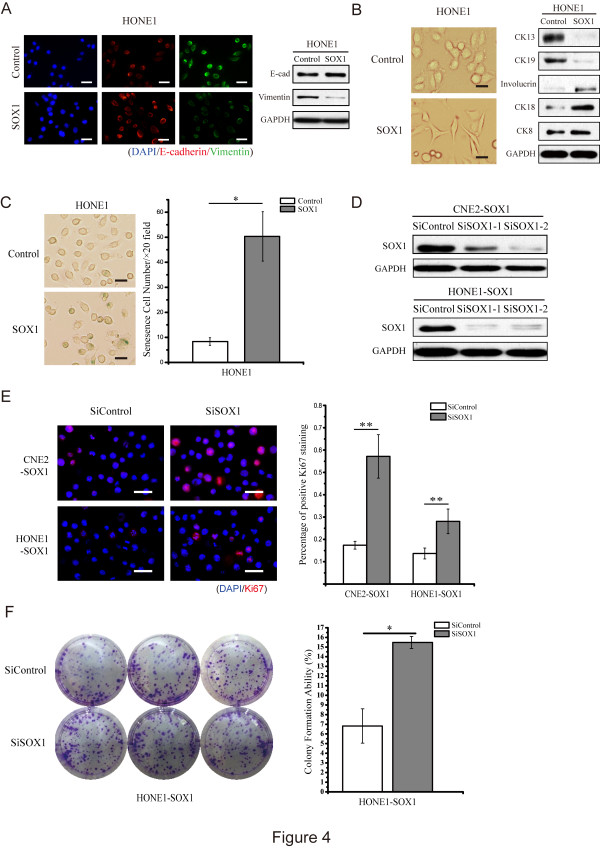
**SOX1 reduces EMT, stimulates cell differentiation and triggers cellular senescence, whereas transient knockdown of SOX1 partially reverses the malignant phenotype. (A)** Presence of the EMT-related proteins E-cadherin and Vimentin was detected via IF and WB. IF (left panel) Blue, DAPI; Red, E-cadherin; Green, Vimentin. EMT-related markers were detected by WB (right panel) in HONE1 cells with forced SOX1 expression. GAPDH served as an internal control. **(B)** Morphology of differentiated HONE1 cells induced by SOX1 was observed under microscopy. WB was used to detect the cell surface markers related to cell differentiation in HONE1 cells with or without SOX1 overexpression. **(C)** HONE1 cells with or without SOX1 overexpression were fixed and stained for SA-β-gal activity. The number of senescent colonies within each 20× field of HONE1 cells increased from 8.33 ± 1.53 to 50.33 ± 9.87. Bar represents mean ± SD of three independent experiments. (*p <0.05, Student’s t test) **(D)** SOX1 expression level in SOX1-overexpressing-NPC cells following transfection of SOX1-specific siRNA was determined using WB. **(E, F)** Ki67 staining rate and colony formation ability following knockdown of SOX1 using siRNA in SOX1-overexpressing NPC cells. Ki67 staining rate increased from 0.17 ± 0.02 to 0.49 ± 0.10 in CNE2 cells and from 0.14 ± 0.02 to 0.28 ± 0.06 in HONE1 cells. Colony formation ability increased from 6.82 ± 1.77% to 15.47 ± 5.28% in HONE1 cells. Bar represents mean ± SD of three independent experiments (*p <0.05, **p <0.01, Student’s t test). (Scale bars, 50 μm in **A**, 25 μm in **B**, **C**, 50 μm in **E**).

The functions of SOX1 *in vitro* were further investigated by overexpressing SOX1 in either CNE2 or HONE1 cells. As shown in Figure 4A and Additional file 2: Figure S2, overexpression of SOX1 down-regulated Vimentin and up-regulated E-cadherin in HONE1 and CNE2 cells, indicating that overexpression of SOX1 suppressed epithelial-mesenchymal transition (EMT). HONE1 is a poorly differentiated NPC cell line, and is histologically characterized by a round and cobblestone-like phenotype. Overexpression of SOX1 in HONE1 cells induced a morphology transition to a slender and fusiform appearance, which was defined as a differentiated phenotype (Figure 4B). This phenotype was similar to those of the well-differentiated NP69 and CNE1 cell lines [26]. Consistent with these morphological changes, expressions of cell differentiation markers such as Involucrin, CK8 and CK18 [27, 28] were increased, whereas undifferentiation markers such as CK19 and CK13 [29, 30] were reduced (Figure 4B). Similar results could be observed in the CNE2 cells (Additional file 1: Figure S1B and S1C). Taken together, these data indicated that SOX1 overexpressing NPC cells were undergoing differentiation. As SOX1 has been implicated in the regulation of cell differentiation and EMT, we further examined the role of SOX1 in stem cell regulation in NPC. We found that sphere-forming ability was dramatically decreased by overexpression of SOX1 in both CNE2 (Additional file 3: Figure S3A, *p* <0.01) and HONE1 (Additional file 3: Figure S3B, *p* <0.01) cells. Meanwhile, colony-forming ability in soft agar was also decreased in these cells (Additional file 3: Figure S3C, both *p* <0.05). We also found that overexpression of SOX1 in HONE1 cells enhanced SA-β-gal staining (Figure 4C).

### Knockdown of SOX1 partially recovers cellular proliferative capacity in SOX1 stably overexpressed NPC cells

To further demonstrate the anti-tumor function of SOX1, we knocked down SOX1 with transient siRNA transfection in CNE2 and HONE1 cells stably overexpressing SOX1. We confirmed SOX1 expression in both cell lines via WB (Figure 4D). Transient knockdown of SOX1 in these cells partially rescued cell proliferation, demonstrated by the increased number of Ki67 (+) cells (Figure 4E) and the enhanced colony formation ability (Figure 4F).

### SOX1 interacts with β-catenin and induces a proteasome-independent down-regulation of β-catenin

**Figure 5:**
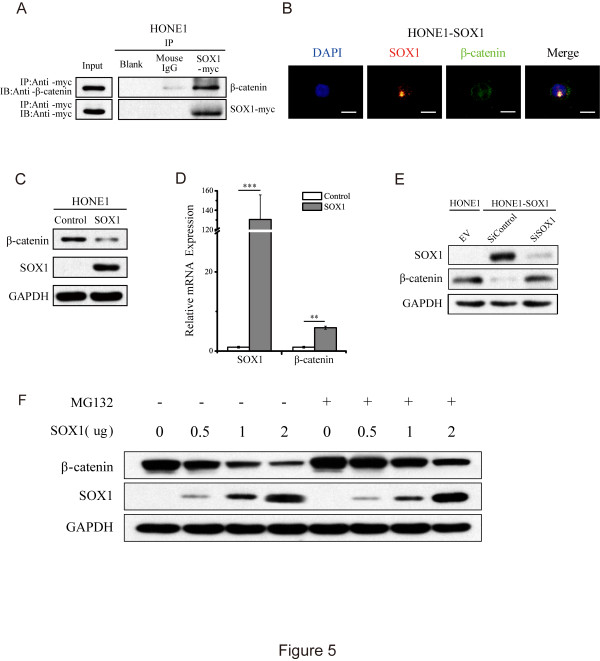
**SOX1 suppresses Wnt/β-catenin signaling by interacting with β-catenin and stimulating its down-regulation in a proteasome-independent manner. (A)** IP was performed on whole-cell lysate from HONE1cells expressing SOX1-myc using an anti-myc-tag antibody. The same amount of mouse immunoglobulin G and blank (empty) were used as negative controls. Immunoprecipitated protein complexes were analyzed by WB to detect β-catenin and SOX1 with myc-tag. **(B)** Merged images depicting co-localization of SOX1 and β-catenin by laser confocal microscopy. Blue, DAPI; Red, SOX1; Green, β-catenin. (Scale bar, 20 μm in B) **(C, D)** The SOX1 and β-catenin protein and mRNA levels of HONE1 cells with or without SOX1 overexpression were detected via WB **(C)** and qRT-PCR **(D)**, (**p <0.01, ***p <0.001, Student’s t test). **(E)** SOX1-overexpressing HONE1 cells were transfected with mock and SOX1 siRNA. SOX1 and β-catenin expression were determined by WB. **(F)** Expression of SOX1 and β-catenin were determined by WB in HONE1 cells transfected with 0, 0.5, 1, or 2 μg of the SOX1 plasmid either with or without the addition of 20 μg/ml MG132 for 12 h. GAPDH was used as an internal control.

Previous studies have shown that SOX1 interacts with β-catenin *in vitro* to suppress β-catenin-mediated TCF/LEF signaling in HCC cell lines [25]. A co-immunoprecipitation assay identified β-catenin and SOX1 in an immunocomplex from HONE1 cell lysate, supporting the existence of this interaction in NPC cell lines (Figure 5A). We also examined the cellular localization of SOX1 and β-catenin using confocal microscopy, and identified SOX1 and β-catenin co-localization in HONE1 cells (Figure 5B). Interestingly, while expression of β-catenin was down-regulated in SOX1-overexpressing HONE1 cells (Figure 5C), transcription of the β-catenin gene was up-regulated (Figure 5D), suggesting that SOX1 reduced expression of β-catenin at the protein level. Furthermore, the down-regulation of β-catenin induced by SOX1-overexpression was reversed by knockdown of SOX1 using siRNA (Figure 5E). Consistently, β-catenin was down-regulated in a dose-dependent manner with increasing amounts of SOX1 (Figure 5F). This phenomenon was not reversed by MG132, a specific proteasome inhibitor, suggesting that SOX1 down-regulated β-catenin in a proteasome-independent manner (Figure 5F).

### SOX1 overexpression modulates the expression of cell cycle-regulating proteins

**Figure 6:**
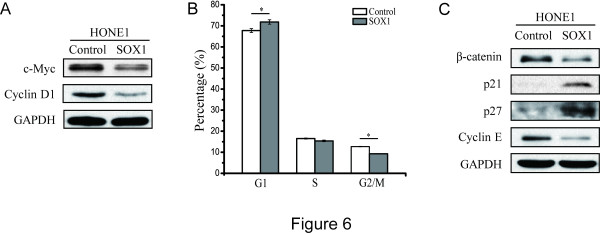
**SOX1 inhibits Wnt/β-catenin downstream genes and modulates the expression of molecules involved in the cell cycle. (A)** WB for expression of c-Myc and Cyclin D1 in SOX1-overexpressing HONE1 cells. **(B)** Flow cytometry cell cycle analysis of HONE1 cells overexpressing SOX1. The percentage of cells in G1 phase increased from 67.77 ± 0.9% to 71.82 ± 1.05% while the percentage of cells in G2/M phase decreased from 12.7 ± 0.10% to 9.3 ± 0.10% in HONE1 cells overexpressing SOX1. Bar represents mean ± SD of three independent experiments. (*p <0.05, Student’s t test). **(C)** Proteins involved in the cell cycle after SOX1 expression was forced in HONE1 cells were identified via WB. GAPDH was used as an internal control.

To further explore the mechanisms by which SOX1 suppresses tumors, we measured the expression of downstream targets of Wnt/β-catenin in SOX1-overexpressing HONE1 cells. A decrease in both c-Myc and Cyclin D1 protein was detected in SOX1-overexpressing HONE1 cells (Figure 6A). To further investigate the influence of SOX1 overexpression on the cell cycle, we evaluated cell cycle progression by flow cytometry. Ectopic expression of SOX1 increased the number of cells in the G1/G0 phase (from 67.77 ± 0.9% to 71.82 ± 1.05%) and decreased the number of cells in the G2/M phase (from 12.7 ± 0.10% to 9.3 ± 0.10%) in HONE1 cells (Figure 6B). To ascertain how SOX1 inhibited cell growth, protein levels of the cell cycle regulators p21, p27 and Cyclin E were examined using WB. In HONE1 cells, SOX1 overexpression significantly enhanced the expression of p21 and p27 but suppressed the expression of Cyclin E (Figure 6C).

## Discussion

These new data provide compelling evidence to suggest that SOX1 suppresses tumorigenicity and regulates expression of β-catenin in NPC. Both NPC cell lines and NPC tissues showed decreased expression of SOX1 at the mRNA and protein levels. Our data further indicate that the decreased expression of SOX1 could be attributed to promoter hypermethylation. Moreover, overexpression of SOX1 in NPC cells reduced tumor formation and the tumor burden *in vivo.* We also found that SOX1 induced NPC cell differentiation and reduced EMT. Mechanistically, SOX1 inhibited the Wnt/β-catenin signaling pathway by promoting β-catenin down-regulation in a proteasome-independent manner. Our study therefore reveals a novel mechanism by which SOX1 regulates tumorigenesis by interfering with Wnt/β-catenin signaling in NPC.

A previous study has shown that another member of the SOX family, SOX11, can also act as a tumor suppressor in NPC cells. Down-regulation of SOX11 mRNA expression was observed in NPC tissues that displayed methylation of *SOX11* promoters. CNE2 cells treated with the hypomethylating agent 5-Aza-CdR recovered expression of SOX11 and experienced inhibited cell growth and invasion [31]. Consistently, we found that SOX1 was also down-regulated via promoter hypermethylation in NPC (Figure 1C and D). Overexpression of SOX1 subsequently decreased expression of β-catenin and suppressed the Wnt/β-catenin signaling pathway, thereby reversing the malignant phenotype of NPC cells. Our findings suggest that an inhibitor of promoter hypermethylation that specifically targets tumor suppressor genes could be beneficial for future NPC treatment strategies.

Functional analysis of the SOX family as transcription factors has demonstrated their important roles in stem cell regulation [8]. These discoveries have linked SOX family proteins with Wnt/β-catenin-mediated regulation of stem cell self-renewal and differentiation [21]. For instance, SOX3 and SOX17 were first reported to regulate the canonical Wnt/β-catenin signaling pathway in *Xenopus* embryos [32]. More recently, the link between SOX family proteins and Wnt/β-catenin signaling has been implicated in tumorigenesis [14, 23]. For example, SOX7 and SOX17 have been suggested to suppress tumor growth through an interaction with β-catenin in various cancer types. Furthermore, overexpression of SOX1 attenuated β-catenin-mediated TCF/LEF signaling via binding with β-catenin and competition for TCF/LEF binding sites in HCC [25].

Previous reports showed that SOX1 acted as a tumor suppressor by interacting with β-catenin in cervical and liver cancers [25, 33]. SOX1 interfered with Wnt/β-catenin signaling by competing for TCF/LEF binding sites in HCC. Consistently, our results demonstrate that SOX1 can suppress malignant properties and induce decreased expression of β-catenin in NPC (Figures 2, 3, 4 and 5C). In the canonical Wnt/β-catenin signaling pathway, β-catenin degradation occurs predominantly through a multiprotein destruction complex comprising factors including tumor suppressors adenomatous polyposis coli (APC), Axin, the kinases casein kinase 1 (CK1) and glycogen synthase kinase 3β (GSK-3β). APC recruits β-catenin to the destruction complex, where it is phosphorylated at N-terminal serine and threonine residues by CK1 and GSK-3β, leading to its ubiquitination and subsequent proteasomal degradation [34]. In the present study, we found that levels of β-catenin protein, but not mRNA, were down-regulated by SOX1 overexpression in a dose-dependent manner (Figure 5F). Furthermore, the down-regulation of β-catenin induced by SOX1 could not be reversed by MG132, suggesting that SOX1 promoted the down-regulation of β-catenin through a proteasome-independent pathway. We also found that SOX1 and β-catenin were present in the same immunocomplex and also co-localized in HONE1 cells (Figure 5A and B). The co-localization of SOX1 and β-catenin partially overlaps with the nuclear DNA signal, suggesting that the co-localization may reside in the cytoplasm and that SOX1 can induce down-regulation of β-catenin in the cytoplasm. These results are consistent with previous reports that β-catenin is degraded in the cytoplasm. Here, we have shown for the first time that SOX proteins can attenuate Wnt/β-catenin signaling independent of the proteasome pathway, and will further investigate the mechanism(s) underlying this SOX1-mediated β-catenin down-regulation in future studies.

Dysregulation of the cell cycle contributes to the etiology of several diseases, including cancer. It has been well established that the Wnt/β-catenin signaling pathway is involved in cell cycle regulation in various cancers [21, 35]. Moreover, as downstream targets of the Wnt/β-catenin signaling pathway, Cyclin D1 and c-Myc play important roles in cell cycle regulation. We found that overexpression of SOX1 decreased β-catenin in NPC cells, and that down-regulation of Cyclin D1 and c-Myc as well as up-regulation of p21 and p27 also occurred in SOX1-overexpressing HONE1 cells (Figure 6A and C). Meanwhile, ectopic expression of SOX1 in HONE1 cells increased the number of cells in the G1/G0 phase while decreasing the number of cells in the G2/M phase (Figure 6B). These results suggested that SOX1 inhibited cell cycle progression by attenuating Wnt/β-catenin signaling. Additional evidence of this is supplied by studies of neural system development, where SOX1 binding to β-catenin suppressed β-catenin-mediated TCF/LEF signaling, thereby promoting cell cycle exit [36]. Further studies are needed to investigate the mechanism involved in the cell cycle regulation by SOX1.

Previous reports have demonstrated that SOX1 regulates the differentiation of neural progenitor cells by directly binding to β-catenin and suppressing β-catenin-mediated TCF/LEF signaling [36, 37]. These reports are consistent with our finding that overexpression of SOX1 triggers differentiation of CNE2 and HONE1 cells, which are both poorly differentiated NPC cell lines (Figure 4B and Additional file 1: Figure S1B and S1C). Further investigation is needed to explore how SOX1 regulates cancer cell differentiation and whether the interaction between SOX1 and β-catenin is involved in this regulation. Our findings will provide new guidance for NPC treatment and improve prognoses in the future.

## Conclusion

SOX1 expression is frequently down-regulated in NPC because of promoter hypermethylation. Overexpression of SOX1 reverses the malignant phenotype of NPC cells by suppressing cell migration and EMT and inducing cell senescence and cell differentiation. The potential mechanism by which SOX1 suppresses Wnt/β-catenin signaling is by down-regulation of β-catenin and subsequent inhibition of downstream gene expression. These findings indicate that SOX1 plays a pivotal role as a tumor suppressor in NPC development.

## Methods

### Cell lines and cell culture

Six NPC cell lines (CNE1, CNE2, HONE1, SUNE1, 58 F and HK1) and an immortalized normal nasopharyngeal epithelial cell line (NP69) were used in this study. All cells were obtained from the State Key Laboratory of Oncology in South China. CNE1 and CNE2 were cultured in RPMI-1640 (Gibco, Life Technologies, CA, USA) containing 10% fetal calf serum (Gibco). HONE1, SUNE1, 58 F and HK1 were cultured in RPMI-1640 containing 10% fetal bovine serum (Hyclone, South America). NP69 cells were propagated in defined keratinocyte-serum-free medium (K-SFM; Gibco, Life Technologies, Basel, Switzerland) supplemented with growth factors. Virus-converted cells with and without stable SOX1 overexpression (CNE2-pLVPT and CNE2-SOX1, HONE1-pLVPT and HONE1-SOX1) were maintained in incomplete medium with 1.5 μg/ml puromycin. All cells were maintained at 37°C in a humidified atmosphere containing 5% CO_2_.

### Bisulfite conversion and quantitative methylation-specific polymerase chain reaction (qMS-PCR)

qMS-PCR was performed using the EZ DNA Methylation-Gold™ Kit (ZYMO Research, CA, USA) according to the manufacturer’s protocol. The primer sequence for qMS-PCR has been described [38]. Methylated primers for each construct were as follows: hSOX1, forward 5′-CGTTTTTTTTTTTTCGTTATTGGC-3′, reverse 5′-CCTACGCTCGATCCTCAACG-3′; unmethylated primers for hSOX1, forward 5′-TGTTTTTTTTTTTTTGTTATTGGTG-3′, reverse 5′-CCTACACTCAATCCTCAACAAC-3′. All qMS-PCR data were obtained from at least three independent experiments to ensure reproducibility.

### RNA isolation, reverse-transcription PCR (RT-PCR) and real-time quantitative RT-PCR

RNA was isolated from each tissue or cell sample using Trizol reagent (Invitrogen, CA, USA) according to the manufacturer’s protocol. A 2-μg aliquot of total RNA from each sample was used for cDNA synthesis with SuperScript® III RT (Invitrogen) and an oligo-dT primer. PCR was performed at 95°C for 5 min, then 30 cycles (95°C for 5 sec, 55–68°C for 15 sec and 72°C for 1 min) with a final extension of 10 min at 72°C. The primer sequence for SOX1 was as follows: forward 5′-AAGGTGAAGGTCGGAGTCAAC-3′, reverse 5′-GGGGTCATTGATGGCAACAATA-3′. Real-time PCR was performed using Platinum® SYBR® Green qPCR SuperMix (Invitrogen) and analyzed on a CFX96 Real-Time System (Bio-Rad, CA, USA). Glyceraldehyde-3-phosphate dehydrogenase gene (GAPDH) was used as an internal control. The difference of real-time PCR cycle number (Ct value) between the target gene and GAPDH was quantified using the ΔΔCT method.

### Western blot (WB) and antibodies

The WB procedure was performed as described previously [39] with some modifications. Briefly, after transient transfection with plasmids, cells were harvested and lysed in a lysis buffer containing a cocktail of protease inhibitors. After centrifugation at 12,000 rpm for 15 min at 4°C, supernatants were collected, mixed with dithiothreitol, and used for WB. The ProteoExtract® Subcellular Proteome Extraction Kit (Millipore) was used for extraction of subcellular fractions following the manufacturer’s protocol. Equal amounts of protein extract were electrophoresed in 10% SDS-PAGE gels and then transferred to nitrocellulose membranes. The membranes were blocked with 5% bovine serum albumin (BSA) at room temperature (RT) for 1 h, incubated with the primary antibody overnight at 4°C, and incubated with the secondary antibody at room temperature for 1 h. Blots were washed in Tris-buffered saline with 0.1% Tween-20 and proteins were visualized by chemiluminescence. The following antibodies were used for WB: anti-SOX1 (IB 1:2000, Epitomics, CA, USA), anti-CK19 (1:8000, Epitomics), anti-CK18 (1:4000, Epitomics), anti-CK13 (1:4000, Epitomics), anti-CK8 (1:4000, Epitomics), anti-Involucrin (1:4000, Abcam, MA, USA), anti-GAPDH (1:10000, ProteinTec Group, IL, USA), anti-c-Myc (N-262) (1:2000, Santa Cruz Biotechnology, CA, USA), and anti-β-catenin (1:2000, Upstate, NY, USA).

### Immunofluorescence (IF)

The IF procedure was performed as described previously [40] with some modifications. Briefly, cells were seeded on slides at 50% confluency in 6-well plates for 24 h before fixation. All the following procedures were performed at room temperature. Cells on slides were fixed with 2% formalin for 15 min. After rinsing with phosphate-buffered saline (PBS), slides were permeabilized in 0.25% Triton-X-100 buffer for 15 min. After rinsing with PBS, non-specific sites on the slides were blocked with 3% BSA for 20 min. Slides were then incubated with primary antibodies diluted in 3% BSA for 1 h: anti-SOX1 (1:200), β-catenin (1:200), Ki67 (1:200, Santa Cruz Biotechnology). After rinsing with PBS, the slides were incubated with secondary antibody (1:200) for 1 h in the dark. The slides were rinsed with PBS, stained with 100 ng/ml 4′,6-diamidino-2-phenylindole for 5 min, and rinsed with PBS. The slides were dried and placed on embedding medium on cover slides, and then observed under a fluorescence microscope.

### Immunoprecipitation

Immunoprecipitation was conducted according to a previous report [26]. Polyclonal Anti-c-myc-tag (Sigma-Aldrich, MO, USA) antibody was cross-linked to protein G beads (Invitrogen) according to the manufacturer’s protocol. Cell lysate from NPC cells transfected with SOX1-myc plasmid was pre-cleared with IgG beads-protein G for 2 h and then incubated with c-myc-tag-beads overnight at 4°C. C-myc-tag-immunoprecipitated complexes were washed five times with immunoprecipitation buffer (10 mM Tris/HCl, pH 7.8; 1 mM EDTA; 150 mM NaCl; 1 mM NaF; 0.5% Nonidet P-40; 0.5% glucopyranoside; 1 μg/ml aprotinin; and 0.5 mM phenylmethylsulfonyl fluoride). Proteins were eluted from beads by boiling in loading buffer, and then subjected to WB. Anti-β-catenin and anti-c-myc-tag antibodies were used to detect β-catenin and SOX1-myc.

### Colony formation assay

The colony formation assay was performed according to the procedure reported previously [41]. Briefly, cells were trypsinized, resuspended as single cells and plated onto 6-well plates with 500 cells per well. After 10 days, the colonies were fixed with 2% methanol and stained with crystal violet. Colonies with more than 50 cells were counted under the microscope. The cloning efficiency was calculated using the whole area of the colonies.

### Wound-healing assay and transwell migration assay

For the wound-healing assay, CNE2-EV&SOX1 and HONE1-EV&SOX1 cells were seeded in a 6-well plate at 90% confluence. The day before the assay, cells were starved with serum-free medium overnight. A sterile 200-μl pipet tip was used to scratch three separate wounds through the cells moving perpendicular to the line drawn with a marker on the bottom of the dish. Then, cells were gently rinsed with PBS and 1.5 ml of media was added. Pictures were taken using a phase contrast microscope at 0, 12, 16 and 20 h. For the transwell migration assay, 800 μl of normal medium was added to the lower chamber in a 24-well plate, and 200 μl cell suspension (1 × 10^5^ cells) in SFM was added to the upper chamber. After incubation for 24 h (37°C, 5% CO_2_), the membrane was fixed with 2% methanol and stained with crystal violet. Pictures were taken under a microscope and the stained cells in the lower side of the membrane were counted.

### *In vivo*tumorigenicity

CNE2 cells (5 × 10^5^) transfected with empty vector or SOX1-expressing plasmid were injected subcutaneously into the left and right flanks of 6-week-old nude mice (Guangdong Medical Laboratory Animal Center, Guangdong, China). The tumor volume was calculated as 0.5 × L1 × (L2)^2^, where L1 is the long axis and L2 is the short axis of the tumor. The developing tumors were observed every third day. After 20 days, the mice were sacrificed at the end of follow-up. The animal study protocol was approved by the Institutional Animal Care and Use Committee.

### Senescence-associated β-galactosidase staining

CNE2-EV&SOX1 and HONE1-EV&SOX1 cells were plated in a 6-well plate. The Senescence-β-Gal Staining Kit (Beyotime Institute of Biotechnology, Jiangsu, China) was used according to the manufacturer’s instructions. Briefly, cells were fixed with fixation buffer for 10 min after rinsing three times in PBS for 5 min at RT. The cells were stained with staining buffer at 37°C overnight covered with plastic wrap. The cells were rinsed three times in PBS for 5 min at RT and observed under a microscope.

### Flow cytometry for cell cycle analyses

Cells were harvested by digestion, washed with pre-chilled PBS, fixed with 70% ethanol at 4°C for 30 min, then stained with propidium iodide (Sigma-Aldrich, 50 mg/ml) for 30 min at 4°C in the dark. Stained cells were subjected to flow cytometry analysis to measure the sub-G1 fraction.

### Sphere formation assay

Sphere formation assays were performed as per a previous report [42]. Cells were seeded into an ultralow attachment 24-well plate (Corning) at 500 cells per well. Cells were grown in a DMEM/F12 medium (Gibco), supplemented with B27 (Invitrogen), EGF (20 ng/ml), bFGF (20 ng/ml, BD Biosciences), and heparin (0.5 U/ml, Sigma). Fresh medium was added (200 μl/well) and the plates were gently shaken every other day. Spheres were counted following 10–15 days of culture.

### Soft agar colony formation assay

Soft agar colony formation assays were conducted according to a previous report [43]. Briefly, cells were resuspended in medium containing 0.6% agarose (500 cells/well) and seeded into a 6-well plate coated with 1.2% agarose. Then 2 ml culture medium was added on the top to maintain moisture and nutrient availability. Colonies were counted after culture for 2–3 weeks.

### Statistical analysis

Statistical analyses were performed using the SPSS software, version 16.0 (SPSS Inc.) or with GraphPad Prism 5.0 (GraphPad Software, Inc.). The unpaired Student’s t test was used to perform a statistical comparison between two groups. The ANOVA test was used when performing multiple comparisons. The level of significance was set at *p* <0.05. Error bars represent the standard deviation of the mean (SD), and each experiment was completed at least twice with samples in triplicate.

## Electronic supplementary material

Additional file 1: Figure S1: Ectopic expression of SOX1 represses CNE2 cells migration and induces cell differentiation *in vitro*. (A) The wound-healing assay performed in CNE2 cells overexpressing SOX1. (B) Morphology of differentiated CNE2 cells induced by SOX1 was observed under microscopy. (C) WB was used to detect the cell surface markers related to cell differentiation in CNE2 cells with or without SOX1 overexpression. (Scale bars, 200 μm in A and 25 μm in B). (TIFF 7 MB)

Additional file 2: Figure S2: SOX1 reduces epithelial–mesenchymal transition in CNE2 cells. (A) Presence of the EMT-related proteins E-cadherin and Vimentin were detected via IF in CNE2 cells. Blue, DAPI; Red, E-cadherin; Green, Vimentin. (B) EMT-related markers were detected by WB in CNE2 cells with forced SOX1 expression. GAPDH served as an internal control. (TIFF 2 MB)

Additional file 3: Figure S3: Overexpression of SOX1 negative regulated the stem cell ability of NPC cells. (A, B) The sphere formation ability reduced significantly in both CNE2 (from 64.5 ± 9.95 to 32 ± 10.23 per 500 cells, **p < 0.01, Student’s t test) and HONE1 (from 31.25 ± 5.12 to 12.75 ± 3.50 per 500 cells, **p < 0.01). (C) The colony formation ability in soft agar dramatically decreased upon SOX1 overexpression, from 19.3 ± 4.5 to 5.3 ± 1.5 each 20× field in CNE2 and from 20.7 ± 5.0 to 7.7 ± 2.1 each 20× field in HONE1, both *p < 0.05, Student’s t test. Quantitative data were shown as the mean ± SD from three independent experiments. (Scale bars, 100 μm in A, B, 1000 μm in C). (TIFF 9 MB)

## Abbreviations

BSA: Bovine serum albumin
CSC: Cancer stem cell
EMT: Epithelial-mesenchymal transition
HCC: Hepatocellular carcinoma
HMG: High-mobility group
IP assay: Immunoprecipitation assay
LEF: Lymphoid enhancer factor
NPC: Nasopharyngeal carcinoma
PBS: Phosphate-buffered saline
PCR: Polymerase chain reaction
qMS-PCR: Quantitative methylation-specific PCR
RT-PCR: Quantitative real-time PCR
SPARC: Secreted protein, acidic, cysteine-rich
TCF: T-cell factor.
