# Supplementary figures and images for: SOX1 down-regulates β-catenin and reverses malignant phenotype in nasopharyngeal carcinoma
Source: Mol Cancer. 2014 Nov 26;13:257. doi: 10.1186/1476-4598-13-257 (PMC4326525; doi:10.1186/1476-4598-13-257)

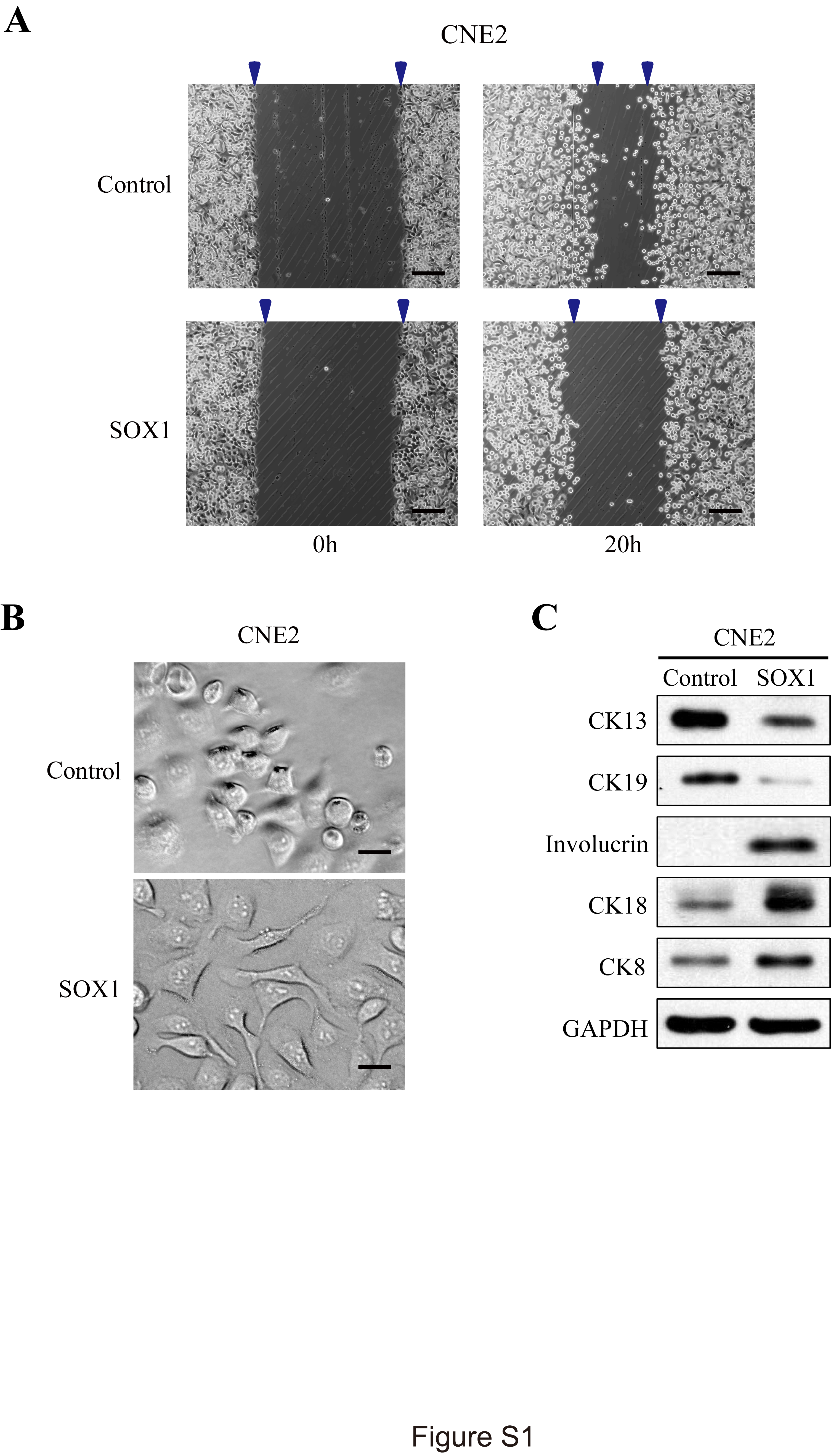

Supplement: Supplementary file 1 — Additional file 1: Figure S1: Ectopic expression of SOX1 represses CNE2 cells migration and induces cell differentiation in vitro. (A) The wound-healing assay performed in CNE2 cells overexpressing SOX1. (B) Morphology of differentiated CNE2 cells induced by SOX1 was observed under microscopy. (C) WB was used to detect the cell surface markers related to cell differentiation in CNE2 cells with or without SOX1 overexpression. (Scale bars, 200 μm in A and 25 μm in B). (TIFF 7 MB) [file 12943_2014_1470_MOESM1_ESM.tiff]

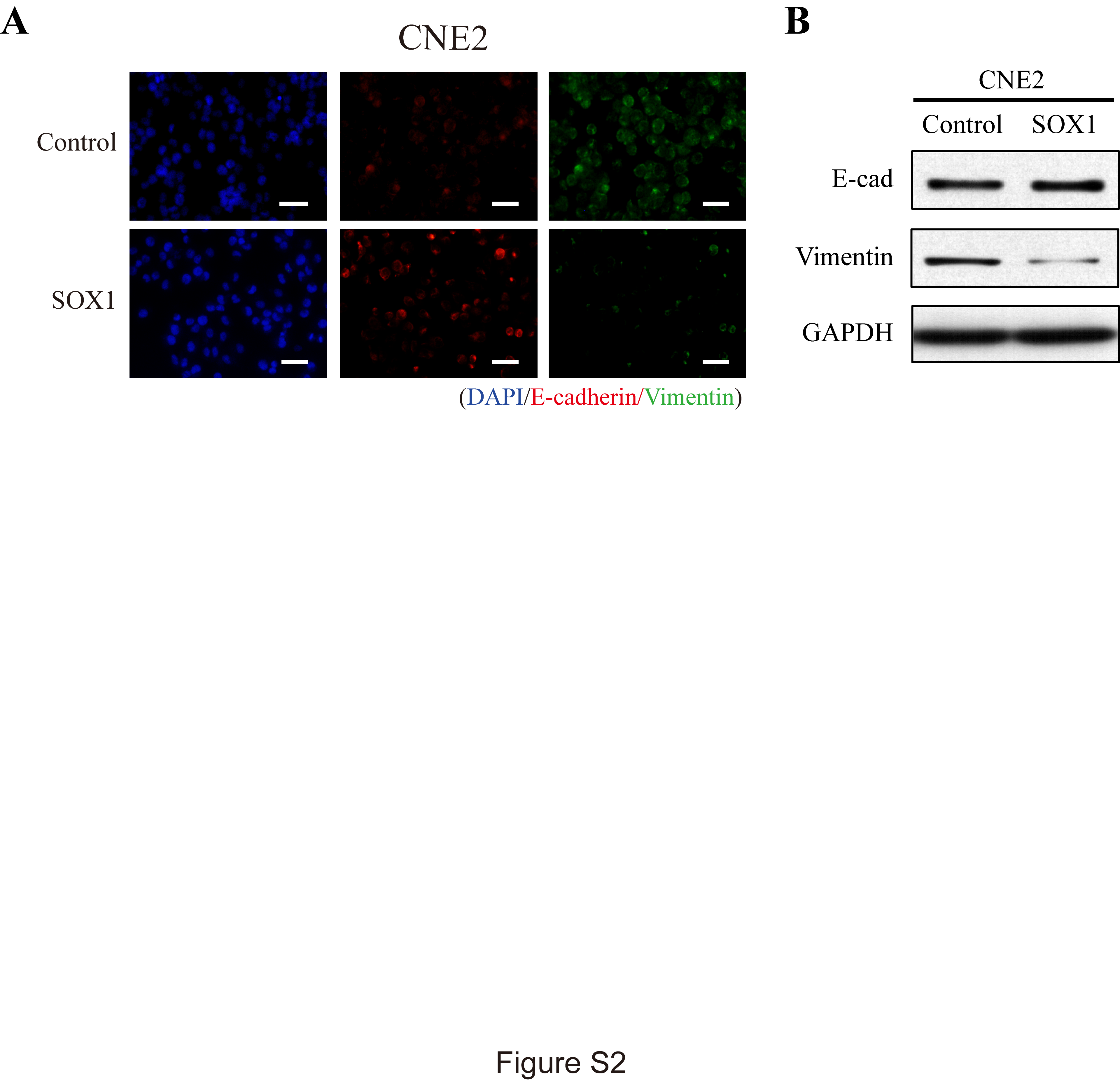

Supplement: Supplementary file 2 — Additional file 2: Figure S2: SOX1 reduces epithelial–mesenchymal transition in CNE2 cells. (A) Presence of the EMT-related proteins E-cadherin and Vimentin were detected via IF in CNE2 cells. Blue, DAPI; Red, E-cadherin; Green, Vimentin. (B) EMT-related markers were detected by WB in CNE2 cells with forced SOX1 expression. GAPDH served as an internal control. (TIFF 2 MB) [file 12943_2014_1470_MOESM2_ESM.tiff]

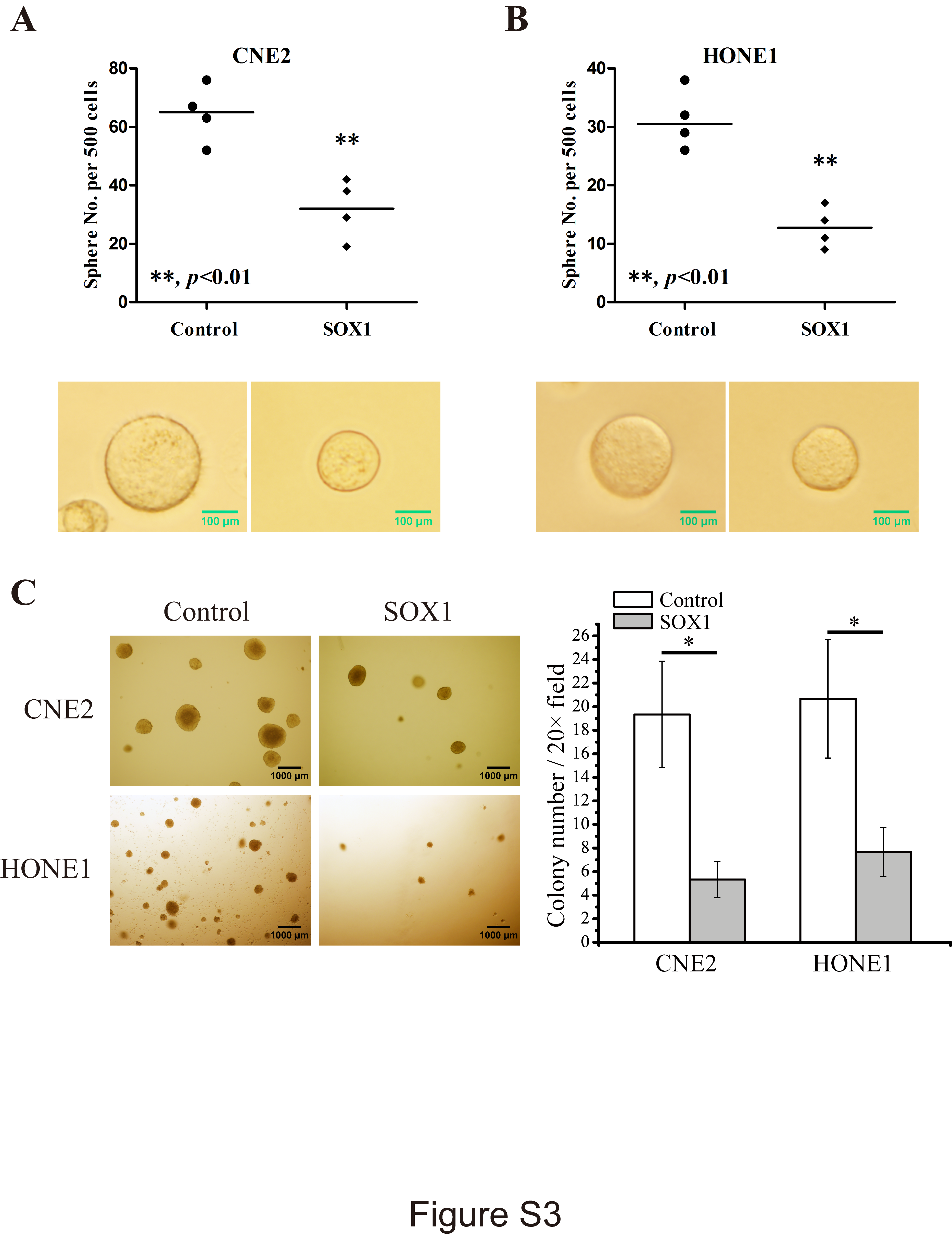

Supplement: Supplementary file 3 — Additional file 3: Figure S3: Overexpression of SOX1 negative regulated the stem cell ability of NPC cells. (A, B) The sphere formation ability reduced significantly in both CNE2 (from 64.5 ± 9.95 to 32 ± 10.23 per 500 cells, **p < 0.01, Student’s t test) and HONE1 (from 31.25 ± 5.12 to 12.75 ± 3.50 per 500 cells, **p < 0.01). (C) The colony formation ability in soft agar dramatically decreased upon SOX1 overexpression, from 19.3 ± 4.5 to 5.3 ± 1.5 each 20× field in CNE2 and from 20.7 ± 5.0 to 7.7 ± 2.1 each 20× field in HONE1, both *p < 0.05, Student’s t test. Quantitative data were shown as the mean ± SD from three independent experiments. (Scale bars, 100 μm in A, B, 1000 μm in C). (TIFF 9 MB) [file 12943_2014_1470_MOESM3_ESM.tiff]
